# Promoting effects of soil C and N and limiting effect of soil P jointly determine the plant diversity during the aerial seeding restoration process in Mu Us sandy land, China

**DOI:** 10.3389/fpls.2023.1272607

**Published:** 2023-10-25

**Authors:** Xiaoqian Gong, Zhimin Qi, Jia Wen, Yongzhi Yan, Qingfu Liu, Yuanheng Li, Qing Zhang

**Affiliations:** ^1^ Ministry of Education Key Laboratory of Ecology and Resource Use of the Mongolian Plateau, School of Ecology and Environment, Inner Mongolia University, Hohhot, China; ^2^ School of Life Sciences, Inner Mongolia University, Hohhot, China; ^3^ Research Center of Forest Ecology, Forestry College, Guizhou University, Guiyang, China; ^4^ Institute of Grassland Research, Chinese Academy of Agricultural Sciences, Hohhot, China; ^5^ Collaborative Innovation Center for Grassland Ecological Security (Jointly Supported by the Ministry of Education of China and Inner Mongolia Autonomous Region), Inner Mongolia University, Hohhot, China

**Keywords:** ecological restoration, soil nutrients, functional diversity, phylogenetic diversity, taxonomic diversity

## Abstract

**Introduction:**

Exploring the change and maintaining mechanism of plant diversity is of great significance for guiding the restoration of degraded ecosystems. However, how plant taxonomic, functional, and phylogenetic diversity change during long-term ecosystem restoration process and their driving factors remain unclear.

**Methods:**

Based on the 35-year time gradient of aerial seeding restoration in Mu Us sandy land, this study explored the changes in plant taxonomic, functional, and phylogenetic diversity and the driving factors.

**Results:**

The results showed that plant taxonomic, functional, and phylogenetic diversity showed consistent response with the aerial seeding restoration, all of which increased first and then tended to a saturation state in the middle of restoration (14 years). TN, TOC, and NO_3_^-^-N increased with aerial seeding restoration and showed a significant positive correlation with plant diversity of the three dimensions, while AP showed a negative correlation. Soil nitrogen and carbon promoted the increase of diversity of three dimensions in the early restoration period, while phosphorus limited the increase of diversity of three dimensions in the middle and late restoration periods. The diversity of three dimensions was mainly affected by restoration time, soil nutrients, and climate factors, and the coupling effect of restoration time and soil nutrients was dominant.

**Discussion:**

These findings indicate that the plant diversity in different dimensions and soil nutrients are improved by aerial seeding restoration. Our study highlights that aerial seeding restoration mainly improves plant diversity by increasing soil nutrients, and the relative effects of different soil nutrients on plant diversity during restoration are inconsistent.

## 1 Introduction

Ecosystem degradation can lead to species extinction and biodiversity loss (Marco et al., 2019). As a fundamental ecosystem component, biodiversity plays an important role in maintaining ecosystem functions and services (Oliver et al., 2015). In 2019, the Intergovernmental Science-Policy Platform on Biodiversity and Ecosystem Services released a report showing that one million species of plants and animals are endangered due to human-induced ecological degradation (Díaz et al., 2019). Ecological restoration can help reverse the degradation trend, increase biodiversity, and improve ecosystem health (Montoya, 2021). A recent study has shown that ecological restoration increased biodiversity by an average of 20% at 83 restoration sites worldwide (Atkinson et al., 2022). The impact of ecological restoration on biodiversity has become a hot topic in current ecological research.

Taxonomic diversity, which reflects taxonomic information of species, is commonly used to assess the effect of ecological restoration on biodiversity (O’brien et al., 2022; Yan et al., 2022). However, biodiversity generally has three dimensions, including taxonomic, functional, and phylogenetic diversity. Although functional and phylogenetic diversity are also important parts of biodiversity, they have received less attention in ecological restoration. Functional diversity reflects the differences in functional traits among species in communities (Qin et al., 2016). Functional traits are the functional attributes closely related to the growth, reproduction, and competition of species and can more directly reflect the competitive relationship and resource utilization of species (Yan et al., 2020). Phylogenetic diversity represents the diversity of evolutionary differences among species in communities. Functional and phylogenetic diversity can complement taxonomic diversity and describe biodiversity from functional and evolutionary perspectives (Qin et al., 2016). Exploring taxonomic, functional, and phylogenetic diversity can more comprehensively inform the ecological restoration effect.

Biodiversity is affected by many factors during the ecological restoration process, including soil and climate factors (Aguirre-Gutiérrez et al., 2020; Shi et al., 2020). Different types of soil nutrients have different effects on biodiversity. The study found that soil carbon and nitrogen content increased with forest restoration, while phosphorus became a limiting factor for plant growth (Yang et al., 2021). As the two most concerned factors in climate, temperature, and precipitation also have different impacts on biodiversity. A study on the Qinghai-Tibet Plateau found that warming decreased plant diversity, while precipitation increased plant diversity (Wang et al., 2022). Determining the key factors affecting biodiversity is significant for understanding the changes and driving mechanisms of biodiversity during the ecological restoration process.

In addition, the responses of biodiversity on three dimensions to driving factors differ. Previous studies have found that taxonomic diversity is closely related to soil factors during the restoration process, including soil carbon and nitrogen (Zhao et al., 2015). However, taxonomic, functional, and phylogenetic diversity reflect different aspects of diversity, and their influencing factors may differ (Barber et al., 2019; Shi et al., 2020). Some studies have found that functional diversity is affected by both soil and climate factors, while phylogenetic diversity is mainly affected by climate factors (Shi et al., 2020). Therefore, it is of great significance to explore the influencing factors of diversity in the three dimensions simultaneously to clarify the biodiversity restoration and driving mechanism during the ecological restoration process and thus help guide ecological restoration and biodiversity conservation (Yan et al., 2023). However, the effects of soil and climate factors on biodiversity on three dimensions during restoration are still unclear.

33% of the world’s land is being degraded, affecting the livelihoods of billions of people worldwide (Abhilash, 2021). Desertification is one of the severe consequences of land degradation, and in the context of climate change, desertification continues to increase at the rate of 12 million hectares per year (Sun et al., 2023). China is one of the countries most seriously affected by desertification, with 27.33% of the land area being threatened by desertification (Cheng et al., 2021b). The government has implemented restoration measures to combat desertification, such as the Grain for Green Project and the Three Norths Shelter Forest Program (Liu et al., 2020). Mu Us sandy land is one of the four major sandy lands in China, located in the agro-grazing ecotone, and has always been a key area for Chinese desertification combating (Cheng et al., 2021a). Aerial seeding is a measure of artificial restoration by sowing seeds with aerial equipment, which has achieved remarkable effects in Mu Us sandy land (Gong et al., 2023). Most researchers mainly focus on the plant diversity in a single dimension during the aerial seeding restoration process in Mu Us sandy land (Liu et al., 2021). However, the impact of aerial seeding restoration on plant diversity in different dimensions are still unclear. Therefore, based on a 35-year aerial seeding restoration gradient in Mu Us sandy land, this study explored the changes and driving factors of plant diversity during the aerial seeding restoration process from three dimensions (taxonomic, functional, and phylogenetic diversity). Our objective is to fully reveal the effects of aerial seeding restoration on plant diversity and provide theoretical support for the assessment of aerial seeding restoration effects in Mu Us sandy land.

## 2 Materials and methods

### 2.1 Study area and sites sampled

**Figure 1:**
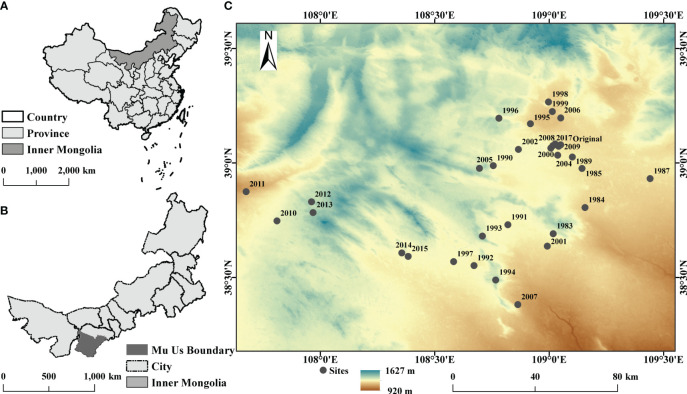
Study area in Mu Us sandy land, Inner Mongolia, China. **(A)** The map of China. **(B)** The map of the Inner Mongolia Autonomous Region. **(C)** The 32 study sites in Mu Us sandy land.

The study area is located in Mu Us sandy land (107°4’–109°26’ E, 38°22’–39°15’ N) in northern China (**Figure 1**). The sandy land has a temperate continental climate, with a mean annual temperature and a mean annual precipitation of 6.2°C and 250mm, respectively (Liu et al., 2021). *Artemisia ordosica*, *Salix psammophyla*, and other psammophytes grow on sandy land. The sandy land contains various soil types, such as Kastanozems, Solonchaks, and Histosols (Liu et al., 2021). This area has begun implementing aerial seeding restoration measures since the 1980s, mainly by sowing seeds of *Hedysarum laeve* Maxim and *Hedysarum scoparium*.

We selected 32 sites in Mu Us sandy land, including 30 sites in different years for aerial seeding restoration (1983–2015, excluding 1986, 1988, and 2003), one mobile dune site (2017), and an original site (top community and undisturbed >30 years). We established a 10 m×10 m plot at relatively flat area in each site and set up three 1 m×1 m quadrats along the diagonal at each plot.

### 2.2 Data collection

We conducted the vegetation and soil survey in August 2017 (plant growing season). We investigated, recorded, and collected the plant species in each quadrat and harvested the aboveground biomass of each quadrat by drying it to constant weight in an oven at 65°C. The soil cores were taken at 60 cm depth in each quadrat because precipitation can affect soil moisture up to 60 cm deep (Yu et al, 2018). We thoroughly mixed the samples from the same site, and stored them at 4°C to determine soil nutrient content.

We identified healthy and pest-free individual plants of each species found in sites and determined seven functional traits associated with plant adaptation to drought conditions. Functional traits included plant height (H), leaf area (LA), leaf dry mass (LDM), specific leaf area (SLA), leaf carbon content (LCC), leaf nitrogen content (LNC), and leaf phosphorus content (LPC). H was estimated by measuring the natural vertical height of fifteen individuals of each species. We sampled five whole leaves from each plant and measured LA using a leaf area meter (LI-3100 Area Meter, LI-COR, Lincoln, United States). LDM was obtained by drying and weighing leaves at 65°C. SLA was calculated as the ratio of LA to LDM. We used an elemental analyzer (Euro Vector EA3000; Milan) to determine LCC and LNC and the ammonium molybdate spectrophotometric method to determine LPC (Yan et al., 2019).

Based on the method proposed by Jin and Qian (2022), we obtained the phylogenetic information of species and constructed phylogenetic trees. This method is based on the super phylogenetic tree of 74,531 species of vascular plants worldwide, which can be quickly obtained fine phylogenetic trees in the study. First, we consulted all plant names recorded in the survey using the World Plants (WP) database (https://www.worldplants.de) to obtain recognized species names. Then, we used the ‘phylo.maker’ function in the ‘V.phylomaker2’ package to construct phylogenetic relationships for the species based on the collated list in this study (Jin and Qian, 2022).

We selected two climate factors and six soil nutrient indicators as environmental factors. For climate factors, we selected mean annual temperature (MAT, °C) and mean annual precipitation (MAP, mm), extracted for each site from 30 arc-seconds spatial resolution raster from the WorldClim (v2.1) database (http://www.worldclimate.org/) using ArcGIS software (v10.7) (Fick and Hijmans, 2017). The climate data from the WorldClim (v2.1) database ranged from 1970 to 2000. Moreover, we selected total nitrogen (TN), total organic carbon (TOC), available phosphorus (AP), total phosphorus (TP), nitrate-nitrogen (NO_3_^-^-N), and ammonium nitrogen (NH_4_^+^-N) as six soil nutrient indicators. We measured TN using the Semimicro-Kjeldahl method; TOC using the potassium dichromate heating oxidation method. The AP was determined by the sodium bicarbonate (NaHCO_3_) leaching-Mo-Sb colorimetric method; TP by the alkali fusion-Mo-Sb colorimetric method; NO_3_^-^-N and NH_4_^+^-N by an AA3 continuous flow analytical system (Zhang et al., 2017; Su et al., 2022).

### 2.3 Data analysis

We characterized plant diversity from taxonomic, functional, and phylogenetic diversity. Taxonomic diversity was represented by species richness. Functional diversity was represented by Euclidean distance of function traits among species in the community. Phylogenetic diversity was represented by phylogenetic distance among species in the community.

To investigate the response of plant taxonomic, functional, and phylogenetic diversity to aerial seeding restoration, we used a general linear model to construct a response curve of diversity of three dimensions to aerial seeding restoration time respectively. Previous studies have found that the plant diversity change with the restoration time at the late restoration period showed a threshold effect (Liu et al., 2021). In this study, we used a log-linear model to fit the corresponding curves and calculated the response threshold of plant diversity in three dimensions to aerial seeding restoration time. To explore the response of soil nutrients to aerial seeding restoration, we evaluated the relationship between soil nutrients indicators (including TN, TOC, AP, TP, NO_3_^-^-N, and NH_4_^+^-N) and restoration time using a general linear model.

To explore the relationship between plant taxonomic, functional, and phylogenetic diversity and environmental factors, we used Pearson correlation analysis to calculate the correlation between the plant diversity of three dimensions and soil nutrients and climate factors. Further, to clarify the relative contributions of time, soil nutrients, and climate factors to the diversity of three dimensions during the aerial seeding restoration process, we conducted the variation partitioning analyses to divide the separate and coupled contributions of the three factors affecting the diversity of three dimensions.

All calculations were conducted in R 4.0.5 (R Core Team, 2021). We calculated plant taxonomic, functional, and phylogenetic diversity. Taxonomic diversity was calculated using the ‘vegan’ package (Oksanen et al., 2020), functional diversity based on Euclidean distance of functional traits among species, and phylogenetic diversity based on phylogenetic distance among species was calculated using the ‘picante’ package (Kembel et al., 2010). The ‘lm’ function in the ‘stats’ package was used to curve fit the diversity of three dimensions and restoration time, and the ‘pettitt.test’ function in the ‘trend’ package was used to calculate the response threshold of the diversity of three dimensions to the aerial seeding restoration time (Pettitt, 1979). Soil nutrients and restoration time were fitted to the general linear model by the ‘lm’ function in the ‘stats’ package (R Core Team, 2021). Pearson correlation analysis was carried out based on the ‘cor’ function of the ‘corrplot’ package, and variation partitioning analyses was further conducted based on the ‘varpart’ function of the ‘vegan’ package (Friendly, 2002; Oksanen et al., 2020).

## 3 Results

### 3.1 Change in plant taxonomic, functional, and phylogenetic diversity with restoration time

**Figure 2:**
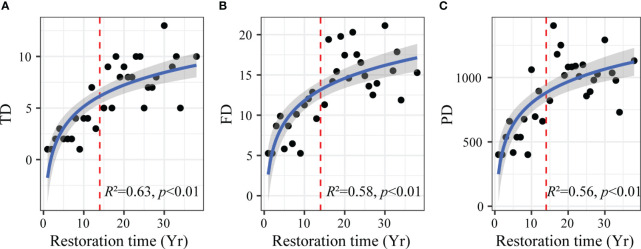
Changes in plant taxonomic **(A)**, functional **(B)** and phylogenetic **(C)** diversity with aerial seeding restoration time. TD, taxonomic diversity; FD, functional diversity; PD, phylogenetic diversity. The red dashed lines indicate the thresholds of taxonomic, functional, and phylogenetic diversity change with aerial seeding restoration time. Yr on the horizontal axis indicates the year.

The log-linear model showed that plant taxonomic (**Figure 2A**), functional (**Figure 2B**), and phylogenetic diversity (**Figure 2C**) showed a similar nonlinear response pattern to aerial seeding restoration time (*p* < 0.01). Plant taxonomic, functional, and phylogenetic diversity increased rapidly in the early restoration period. After 14 years of restoration, the plant diversity of three dimensions increased slowly and gradually reached saturation.

### 3.2 Change in soil nutrients with restoration time

**Figure 3:**
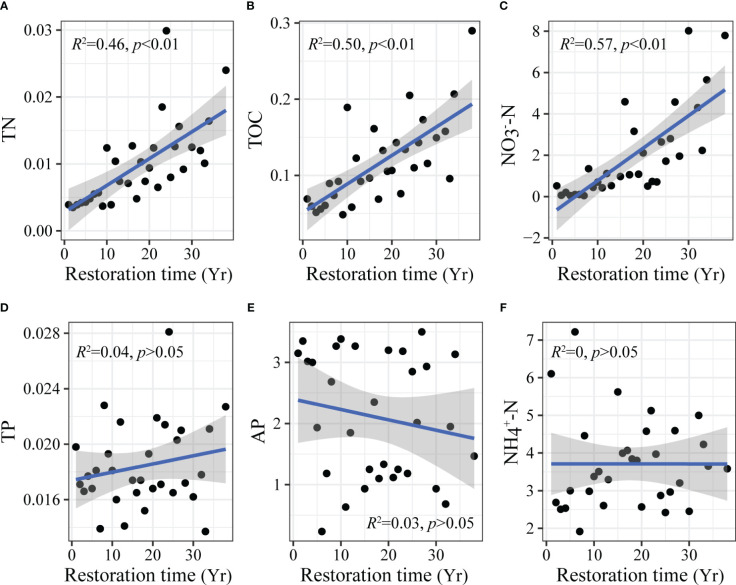
Changes in soil nutrients with aerial seeding restoration time. TN, total nitrogen **(A)**; TOC, total organic carbon **(B)**; NO_3_^-^-N, nitrate-nitrogen **(C)**; TP, total phosphorus **(D)**; AP, available phosphorus **(E)**; NH_4_^+^-N, ammonium nitrogen **(F)**. Yr on the horizontal axis indicates the year.

The general linear model showed that different soil nutrient indicators had different responses to restoration time (**Figure 3**). TN (**Figure 3A**), TOC (**Figure 3B**), and NO_3_^-^-N (**Figure 3C**) showed a highly significant increase with restoration time (*p* < 0.01). TP (**Figure 3D**), AP (**Figure 3E**), and NH_4_^+^-N (**Figure 3F**) had no significant changes (*p* > 0.05).

### 3.3 Correlation of plant taxonomic, functional, and phylogenetic diversity with environmental factors

**Figure 4:**
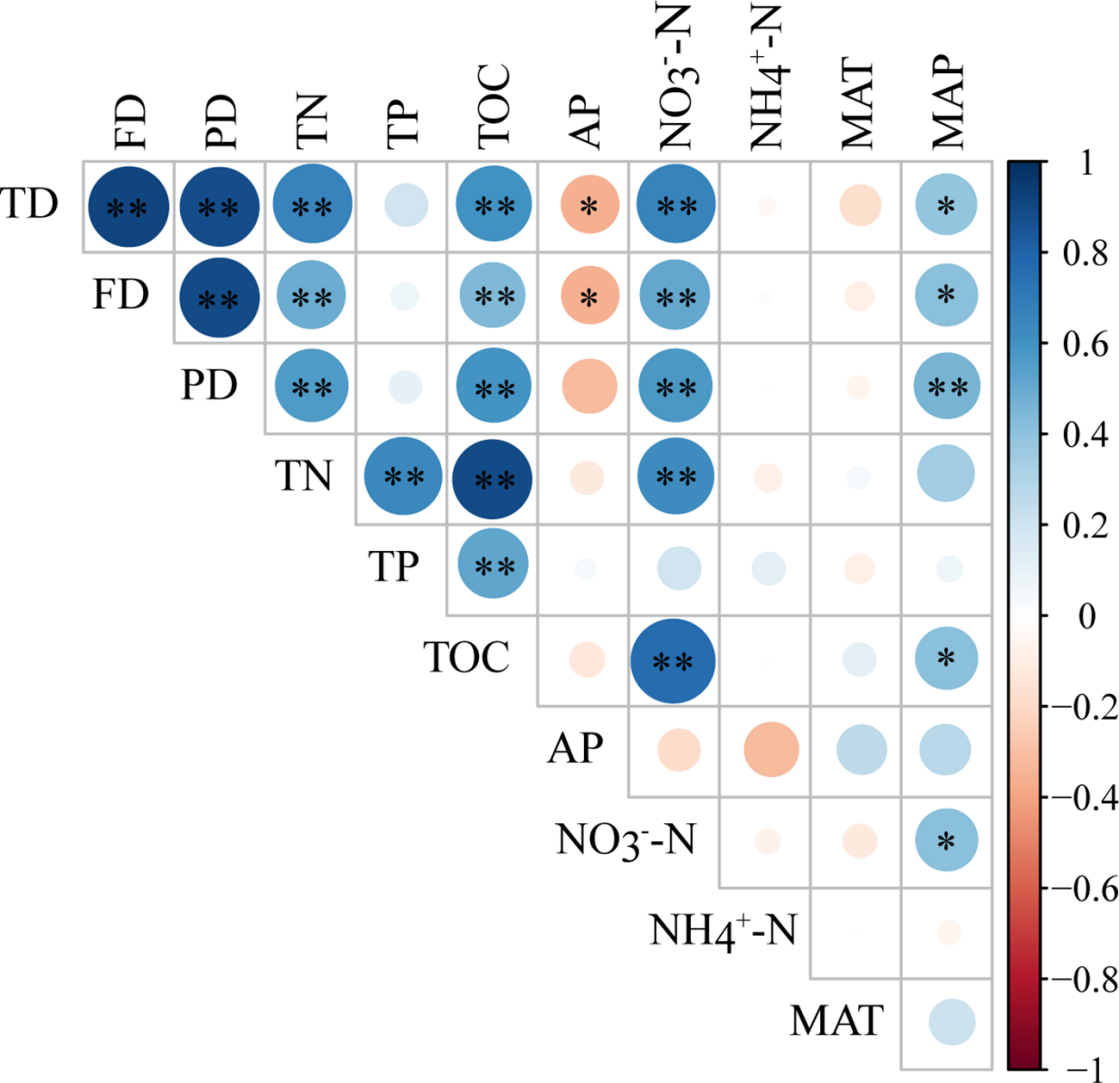
Pearson correlation between plant taxonomic, functional, phylogenetic diversity, and environmental factors. TD, taxonomic diversity; FD, functional diversity; PD, phylogenetic diversity. TN, total nitrogen; TP, total phosphorus; TOC, total organic carbon; AP, available phosphorus; NO_3_^-^-N, nitrate-nitrogen; NH_4_^+^-N, ammonium nitrogen; MAT, mean annual temperature; MAP, mean annual precipitation. * means significant influences at *p* < 0.05 level, and ** means significant influences at *p* < 0.01 level.

Pearson correlation analyses showed that plant taxonomic and functional diversity were significantly positively correlated with MAP (*p* < 0.05), highly significantly positively correlated with TN、TOC、NO_3_^-^-N (*p* < 0.01), and significantly negatively correlated with AP (*p* < 0.05), but had no significant correlation with TP、NH_4_^+^-N and MAT (**Figure 4**). Plant phylogenetic diversity was highly significantly positively correlated with MAP、TN、TOC、NO_3_^-^-N (*p* < 0.01), but had no significant correlation with TP、AP、NH_4_^+^-N and MAT (**Figure 4**).

### 3.4 Relative contributions of restoration time and environmental factors to plant taxonomic, functional, and phylogenetic diversity

**Figure 5:**
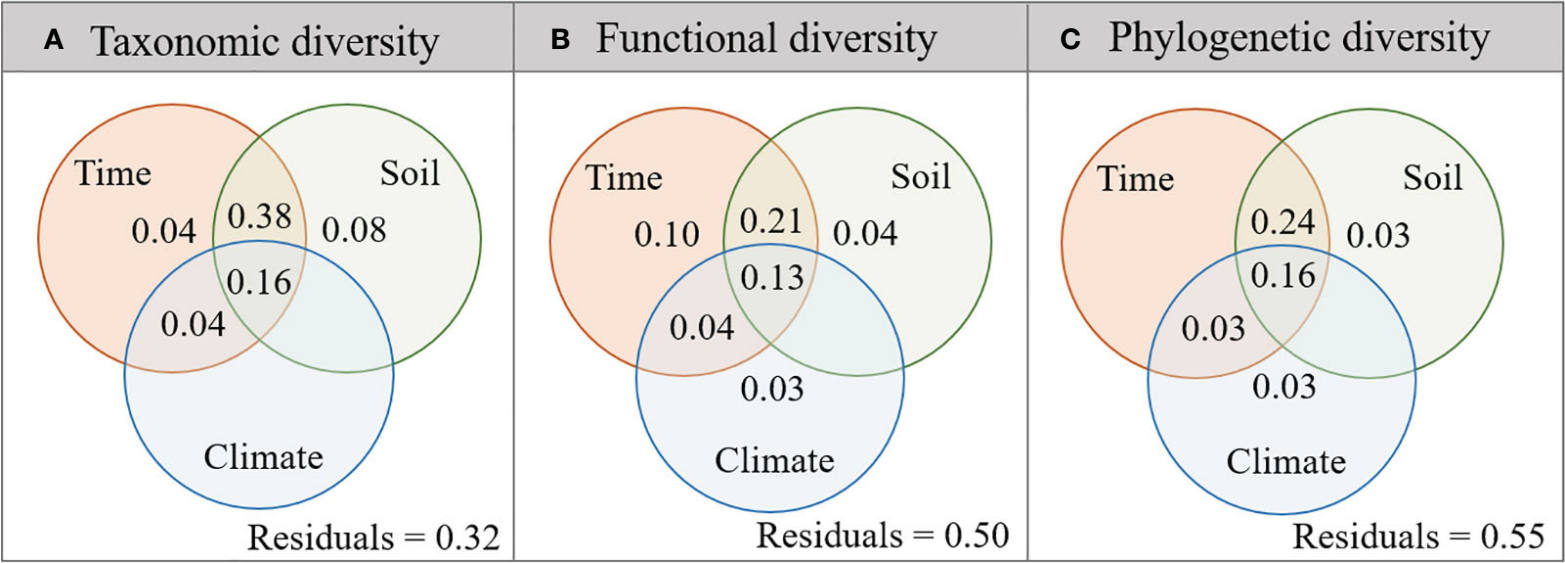
Variance components of plant taxonomic **(A)**, functional **(B)**, and phylogenetic **(C)** diversity by restoration time and environmental factors. The overlap of circles represents the coupling effects of these variable groups.

Variation partitioning analyses showed that plant taxonomic, functional, and phylogenetic diversity was mainly affected by the interaction of restoration time and soil nutrients, which together explained 38% of taxonomic diversity variation (**Figure 5A**), 21% of functional diversity variation (**Figure 5B**), and 24% of phylogenetic diversity variation (**Figure 5C**). In addition, both plant functional and phylogenetic diversity were affected by climate factors (3%), while taxonomic diversity was not significantly affected (**Figure 5**).

## 4 Discussion

### 4.1 The restoration of soil nutrients lags the restoration of plant diversity during the aerial seeding restoration process

Improving soil nutrients help restore degraded ecosystems. Our study found that the aerial seeding restoration significantly increased soil nutrients in Mu Us sandy land (**Figure 3**). TN (**Figure 3A**), TOC (**Figure 3B**), and NO_3_^-^-N (**Figure 3C**) showed a highly significant increase with restoration (*p* < 0.01). The increase of soil carbon and nitrogen with ecosystem restoration was also demonstrated in the study of Yang et al. (2023a). Plant litter, roots, and secretions are important sources of soil nutrients, and more leaf and fine root litter and soil organic matter input caused by vegetation restoration promoted carbon and nitrogen accumulation (Yang et al., 2021). However, this study also found that TP (**Figure 3D**) and AP (**Figure 3E**) did not significantly change with restoration. Soil phosphorus is mainly formed by rock weathering and is affected by soil parent material and biogeochemical processes (Lu et al., 2022). Because the parent material and climate of sandy land were similar during the restoration process, the change of soil phosphorus content was not significant in the short term. Our research showed that soil carbon and nitrogen were restored significantly during the restoration process. However, the restoration of phosphorus is not obvious, which should be paid attention in future restoration.

Exploring changes of plant diversity of three dimensions simultaneously with ecological restoration help to comprehensively understand biodiversity and conservation. Similar to the response of soil nutrients, our study found that plant taxonomic, functional, and phylogenetic diversity increased significantly with aerial seeding restoration (**Figure 2**). This is consistent with previous research, which showed that ecological restoration improved biodiversity in different dimensions (Ribeiro et al., 2018). The reason may be that the soil of sandy land was poor in the early restoration period, plant growth and colonization were difficult, and the taxonomic diversity was low. Moreover, due to strong environmental filtering, the species have near genetic relationships and similar functional traits (Qin et al., 2016). Therefore, plant functional and phylogenetic diversity in the early restoration period was also low. Plant litter and root exudates increased with aerial seeding restoration and improved soil ecological conditions (Wang et al., 2016). The continuous increase of soil nutrients, such as carbon and nitrogen, promoted the colonization and growth of various plants, and the taxonomic diversity increased (**Figure 3**). Meanwhile, the improvement of sandy land resource availability provided a larger shared ecological niche for plants, and provided opportunities for functional traits and phylogenetic differentiation of species, leading to an increase in plant functional and phylogenetic diversity (Galland et al., 2019).

However, we found that in the middle and late restoration period (after 14 years), the plant diversity of three dimensions gradually reached saturation (**Figure 2**), while soil nutrients continued to increase (**Figure 3**). The results showed that the restoration of soil nutrients lagged that of plant diversity during the aerial seeding restoration process. The reason may be that the plant demand for soil nutrients increased with plant diversity in the late restoration period. At this time, plant diversity may be limited by some soil nutrients, such as phosphorus (**Figure 3**). The restriction of soil nutrients can reduce the niche differences among species in the community, leading to the emergence of redundant species with similar functional traits and near genetic relationships, and make the community tend to be stable (Ricotta et al., 2020). Therefore, plant taxonomic, functional, and phylogenetic diversity of species showed saturation trends.

### 4.2 Aerial seeding increase plant diversity mainly by restoring soil nutrients

When considering the relationship between plant taxonomic, functional, and phylogenetic diversity and single factors in soil nutrients and climate during the restoration process, it was found that plant taxonomic, functional, and phylogenetic diversity had a positive relationship with TN, TOC, and NO_3_^-^-N, indicating a positive feedback effect between plant and soil (**Figure 4**). The diverse litter and organic matter contributed by plant can promote the increase of soil C and N content during restoration process (Yang et al., 2021). Functional traits, such as leaf carbon and nitrogen content, may also have significant effect on litter quality and further influence soil decomposition rate and soil nutrients (van Der Putten et al., 2013; Münzbergová and Šurinová, 2015). Moreover, genetic differentiation within species may also impact soil biota and further affect soil communities (Münzbergová and Šurinová, 2015).

On the contrary, soil provides a better environment for nutrient absorption and growth of plants, thus promoting the colonization and growth of different plant species and improving plant diversity from three dimensions through feedback affecting functional trait differentiation and phylogenetic distance (van Der Putten et al., 2013; Münzbergová and Šurinová, 2015). AP was negatively correlated with plant diversity in three dimensions (**Figure 4**). The reason is that phosphorus is the limiting factor of soil nutrients in this area. The higher the plant diversity, the more phosphorus absorption and utilization, and the lower soil phosphorus availability (Zemunik et al., 2015; Yang et al., 2021). Given the different effects of different soil nutrients on plant diversity, species can be selected for the future ecological restoration of Mu Us sandy land, such as planting nitrogen-fixing plants to promote the restoration of sandy land.

Further, this study integrated restoration time, soil nutrients, and climate factors to explore their relative contributions to plant taxonomic, functional, and phylogenetic diversity and found that the plant diversity of three dimensions was mainly affected by the coupling effect of restoration time and soil nutrients during the restoration process (**Figure 5**). The meta-analysis on vegetation ecological restoration conducted by Zhao et al. (2023) on the Loess Plateau found that restoration time was the main factor affecting soil organic carbon after vegetation restoration. This suggested that restoration time improved plant diversity in multiple dimensions, mainly by improving soil nutrients.

In addition, in terms of climate factors, MAP was also significantly positively correlated with plant taxonomic, functional, and phylogenetic diversity (**Figure 4**). A study conducted in the Inner Mongolia grassland also showed that increasing precipitation increased diversity in three dimensions (Hu et al., 2022). Sandy land is located in a semi-arid area, and plants are more sensitive to precipitation change. Increasing precipitation can weaken the environmental filtering caused by water stress and improve the taxonomic diversity by regulating the availability of soil nutrients and water so that the functional traits tend to be diversified (Hu et al., 2022; Yang et al., 2023b). Increased precipitation may also promote the emergence of distant species with different traits through competition, resulting in increased phylogenetic diversity (Yang et al., 2023b).

## 5 Conclusions

This study found that aerial seeding restoration increased plant diversity and soil nutrients in Mu Us sandy land. Plant taxonomic, functional, and phylogenetic diversity significantly improved and began to reach saturation in the middle restoration period. Soil nutrients increased steadily with aerial seeding restoration, and the restoration of soil nutrients lagged behind that of plants. The plant diversity of three dimensions were positively correlated with TN, TOC, and NO_3_^-^-N, and negatively correlated with AP, indicating that soil carbon and nitrogen were the main promoting factors and phosphorus was the main limiting factor for vegetation restoration in Mu Us sandy land. Plant diversity was mainly affected by restoration time, soil nutrients, and climate factors. The coupling effect of restoration time and soil nutrients was dominant. Our study emphasizes that aerial seeding restoration mainly improved plant diversity by increasing soil nutrients, and the effects of soil nutrients on plant diversity are inconsistent during the restoration process.

## Data availability statement

The original contributions presented in the study are included in the article/supplementary material. Further inquiries can be directed to the corresponding authors.

## Author contributions

XG: Writing – original draft, Writing – review & editing, Methodology, Validation, Visualization. ZQ: Methodology, Validation, Visualization, Writing – original draft, Writing – review & editing. JW: Methodology, Validation, Writing – original draft. YY: Methodology, Validation, Writing – original draft. QL: Investigation, Writing – original draft. YL: Conceptualization, Writing – original draft, Writing – review & editing. QZ: Conceptualization, Funding acquisition, Investigation, Methodology, Supervision, Writing – original draft, Writing – review & editing.
